# Dataset on 21 autosomal and two sex determining short tandem repeat loci in the Kedayan population in Borneo, Malaysia

**DOI:** 10.1016/j.dib.2020.105909

**Published:** 2020-06-21

**Authors:** Hashom Mohd Hakim, Hussein Omar Khan, Siti Afifah Ismail, Japareng Lalung, Abban Edward Kofi, Mohd Tajuddin Abdullah, Geoffrey Keith Chambers, Hisham Atan Edinur

**Affiliations:** a DNA Databank Division (D13), Criminal Investigation Department, Royal Malaysian Police, 43200, Cheras, Selangor, Malaysia; b School of Industrial Technology, Universiti Sains Malaysia, 11800, Pulau Pinang, Malaysia; c Forensic Science Programme, School of Health Sciences, Universiti Sains Malaysia, Health Campus, 16150 Kubang Kerian, Kelantan, Malaysia; d Institute of Tropical Biodiversity and Sustainable Development, Universiti Malaysia Terengganu, 21030 Kuala Nerus, Terengganu, Malaysia; e School of Biological Sciences, Victoria University of Wellington, P.O. Box 600, Wellington, New Zealand; f Environmental Futures Research Institute, Griffith University, Nathan, Queensland 4111, Australia

**Keywords:** Genetic markers, Population data, Autosomal str, Forensic parameter, Kedayan and Globalfiler™ express

## Abstract

This data article provides population frequencies for 21 autosomal and two sex determining short tandem repeat (STR) loci in unrelated Kedayan individuals. This article is related to the research paper entitled “Forensic parameters and ancestral fraction in the Kedayan population inferred using 21 autosomal STR loci” [1] where these same data were subjected to ancestry and forensic analyses. We have collected 200 blood samples consisting of 128 male and 72 female volunteer representatives from Kedayan people residing in various parts of Borneo. All 23 STR loci were simultaneously amplified using Globalfiler™ Express PCR and amplicons were separated using an ABI 3500xl Genetic Analyzer. The STR allele calls at each locus were called using GeneMapper^Ⓡ^ ID-X Software v1.4, while several algorithms in Arlequin software version 3.5 were used to estimate Hardy–Weinberg equilibrium (HWE) and linkage disequilibrium (LD) between pairs of STR loci.

Specifications table

**Table:** 

Subject	Genetics
Specific subject area	Forensic genetic markers, DNA analysis, DNA profiling, population genetics, ancestral analyses, human identification
Type of data	Three tables
How data were acquired	Blood samples collected on FTA cards (Whatman, UK) were punched (a single 1.2 mm diameter spot) and mixed with Prep-n-Go™ Lysis Buffer (catalog number: 4,471,406). The reaction mixtures were then subjected to PCR ampilification using Globalfiler™ Express PCR Amplification kit (catalog number: 4,474,665) and followed by capillary electrophoresis. The PCR cycling conditions as per manufacturer's recommendation and previously described [1].
Data format	Raw and analyzed
Parameters for data collection	Allelic variation in 23 STR loci in 200 unrelated, un-admixed and healthy Kedayan individuals
Description of data collection	STR alleles for each locus were called from separated STR amplified products using GeneMapper^Ⓡ^ ID-X Software v1.4. Raw STR data were then compiled in table using Microsoft Office Excel 2013. Allele frequencies were estimated using STRAF software v1.0.5 while HWE &LD analyses were carried out using Arlequin software v3.5.
Data source locations	Sipitang (Longitude 5° 5′ 0″ N and Latitude 115° 33′ 0″ E) and Labuan Island (Longitude 5° 18′ 0″ N and Latitude 115° 13′ 12″ E) in Sabah, Malaysia and Lawas (Longitude 4° 51′ 0″ N and Latitude 115° 24′ 0″ E) in Sarawak, Malaysia
Data accessibility	All data are available within this article
Related research article	[1] H.M. Hakim, H.O. Khan, S.A. Ismail, J. Lalung, A.E. Kofi, M.T. Abdullah, G.K. Chambers, H.A. Edinur, Forensic parameters and ancestral fraction in the Kedayan population inferred using 21 autosomal STR loci. Meta Gene, 25, 100,741. https://doi.org/10.1016/j.mgene.2020.100741

## Value of the data

• The 23 autosomal STR datasets deposited here are the first reported for the Kedayan [2], [3], [4]
• The STR dataset for Kedayan can be used as a reference population in future genetic studies of other ethnic groups in Sabah and Sarawak [5], [6], [7], [8].
• The forensic parameters computed for Kedayan can be used as a standard to properly weight DNA profiles for this ethnic group and their genetically related population groups [9], [10], [11].
• For comparison and validation purposes, the raw STR genotype data Kedayan can be re-analyzed using other algorithms or software [12], [13].

## 1 Data description

In this article we provide a detailed autosomal STR data for Kedayan individuals in Sabah and Sarawak, Malaysia as reported in Hakim et al. [1]. The STR data were obtained by genotyping of 21 autosomal STR loci (D8S1179, D21S11, D7S820, CSF1PO, D3S1358, TH01, D13S317, D16S539, D2S1338, D19S433, VWA, TPOX, D18S51, D5S818, FGA, D12S391, D1S1656, D2S441, D10S1248, D22S1045, SE33 and two sex determining STR loci (Amelogenin and DYS391) in two hundred blood samples of unrelated, un-admixed and healthy individual volunteer representatives (128 males and 72 females) of the Kedayan people. Their STR profiles are shown in Table 1a and Table 1b while Hardy Weinberg equilibrium (HWE) and linkage disequilibrium (LD) values between pairs of 21 autosomal STR loci estimated using Arlequin software version 3.5 are shown in Table 2 and Table 3.

**Table 1a:** STR allelic profiles for AMEL, D3S1358, VWA, D16S539, CSF1PO, TPOX, D8S1179, D21S11, D18S51, DYS391, D2S441 and D19S433 in the Kedayan population (*n* = 200).

Sample ID	Locus											
	ϮAMEL	D3S1358	VWA	D16S539	CSF1PO	TPOX	D8S1179	D21S11	D18S51	ϮDYS391	D2S441	D19S433
A001	Y,X	16,16	18,18	11,11	12,11	11,8	14,13	29,29	19,18	9	12,11.3	14.2,14
A002	Y,X	17,16	18,15	9,9	12,10	8,8	13,13	31,29	20,17	9	11,10	15.2,14
A003	X,X	17,15	17,15	10,9	12,10	11,8	15,14	31.2,29	20,13	–	11,10	15.2,14
A004	X,X	16,15	17,15	10,10	12,12	10,9	16,16	30,29	16,15	–	11.3,11	15,14
A005	Y,X	17,15	18,15	11,9	12,11	9,8	16,13	32.2,30	18,14	11	12,10	13,13
A006	X,X	15,15	18,14	13,9	12,12	9,8	14,14	31,30	16,16	–	10,10	15.2,15.2
A007	Y,X	17,16	18,18	12,9	11,10	11,9	15,14	30,29	23,15	11	11.3,10	13,13
A008	Y,X	17,16	17,15	11,9	12,12	11,8	14,13	30,29	17,16	9	11.3,9.1	14,13
A009	X,X	16,16	17,17	10,10	11,11	11,9	16,16	30,28	15,15	–	11,10	15.2,14.2
A010	Y,X	16,16	18,14	11,10	11,11	11,8	16,13	33.2,30.2	19,16	9	11,9.1	15.2,14
A011	Y,X	17,17	18,14	11,11	12,10	11,8	16,14	30,30	23,13	10	12,10	15.2,13
A012	Y,X	17,17	19,14	13,11	12,10	11,8	15,10	32,29	14,14	9	14,11	13,13
A013	Y,X	17,17	19,15	12,10	12,12	11,8	16,13	32.2,31	17,15	11	11.3,11	15,13
A014	X,X	17,17	19,14	13,12	12,11	8,8	17,16	32.2,32	17,16	–	14,11.3	13,13
A015	Y,X	17,15	18,14	9,9	13,9	11,8	14,13	34.2,30	19,14	9	14,11	15.2,13
A016	Y,X	17,15	18,15	13,11	13,10	9,8	16,10	32.2,30	19,16	10	11.3,11	15.2,14
A017	X,X	16,15	19,14	13,9	10,10	11,8	16,15	31,31	25,18	–	10,10	15,14.2
A018	Y,X	18,17	19,18	13,13	10,9	11,8	16,11	31,30	15,13	11	14,12	15.2,14.2
A019	Y,X	18,17	17,16	13,9	13,12	11,8	16,12	33.2,32.2	23,12	10	12,11	14,13
A020	X,X	17,15	18,17	12,9	12,11	9,8	18,13	32.2,32	18,18	–	12,10	14.2,14
A021	X,X	17,17	18,16	11,9	12,11	11,8	16,11	33,29	23,16	–	11.3,11.3	14,13
A022	X,X	17,16	18,18	13,11	12,11	8,8	18,13	33.2,30	15,14	–	11.3,11	13,13
A023	Y,X	16,15	18,16	10,9	12,12	11,8	16,15	32,30	17,15	10	14,11	14,13
A024	Y,X	17,16	18,16	11,9	12,12	9,8	11,10	34.2,31	18,14	10	11.3,11.3	13,13
A025	Y,X	17,15	17,14	11,9	11,10	11,8	13,10	31,29	14,14	10	14,10	16.2,13
A026	X,X	17,16	18,17	12,11	12,11	9,9	17,14	30,29	17,15	–	14,11	14.2,14.2
A027	Y,X	17,17	17,16	12,9	12,10	11,11	14,14	32.2,31	16,14	11	11.3,10	15.2,14
A028	X,X	17,17	18,17	11,11	12,11	9,8	14,13	32,28	20,17	–	11.3,11.3	14,14
A029	Y,X	17,17	15,14	11,11	12,12	11,8	15,14	30,28	13,12	10	14,14	14.2,14.2
A030	X,X	17,17	19,17	10,9	10,10	11,8	16,12	32,32	17,13	–	14,14	16.2,15.2
A031	Y,X	17,17	18,15	10,9	12,12	11,8	16,13	32,28	15,9	11	14,11.3	14,13.2
A032	X,X	16,15	19,18	11,10	10,9	9,8	18,10	29,29	20,16	–	14,12	13,13
A033	X,X	17,16	18,17	12,9	12,10	11,8	16,16	32,30	19,15	–	11.3,11	15.2,13
A034	Y,X	17,16	18,18	11,9	12,12	11,8	13,13	33.2,29	18,15	10	10,10	13,12
A035	Y,X	17,16	17,14	9,9	11,10	11,8	12,10	31,31	14,14	10	14,9	16.2,15.2
A036	X,X	17,17	17,17	12,9	10,8	9,8	16,14	35.2,30	16,13	–	14,10	14,14
A037	X,X	17,16	18,16	11,9	12,12	11,8	14,13	33.2,32.2	17,14	–	11,10	14,14
A038	X,X	17,17	18,14	11,9	13,12	11,8	16,13	32.2,30	15,14	–	11,11	16.2,14
A039	Y,X	17,15	14,14	12,9	12,12	8,8	15,13	30,29	16,15	10	14,10	14.2,14
A040	X,X	17,17	17,15	10,9	10,10	9,8	14,11	31,29	18,15	–	14,11.3	16.2,14.2
A041	Y,X	17,16	18,16	11,9	12,12	11,8	14,12	33.2,32	17,15	10	11.3,10	14,13
A042	X,X	17,16	18,17	13,9	12,10	11,8	16,15	34.2,32.2	17,16	–	14,10	13.2,13
A043	Y,X	17,16	16,14	11,11	12,11	11,8	13,12	31.2,30	16,15	11	12,11	14.2,14
A044	X,X	16,16	19,14	11,10	11,10	11,8	13,12	32.2,30	15,11	–	13,13	15.2,13.2
A045	Y,X	17,16	18,16	11,11	12,9	11,8	17,13	31.2,30	18,14	10	13,11.3	16.2,15.2
A046	Y,X	16,16	18,16	14,11	11,10	11,8	14,13	33.2,31	17,15	10	11.3,10	16.2,15
A047	Y,X	17,15	15,14	11,10	12,12	11,8	15,12	32,28	12,12	11	14,10	14.2,14
A048	Y,X	17,16	18,17	12,11	12,10	9,8	13,10	31.2,27	13,11	10	14,11	15.2,13
A049	Y,X	15,14	16,14	13,13	12,11	8,8	15,11	32,30	17,15	10	13,11.3	14.2,14.2
A050	Y,X	17,17	15,15	11,9	12,12	11,8	16,11	32,29	20,15	11	14,11.3	14,13.2
A051	X,X	16,16	18,17	11,9	12,10	9,8	14,10	31,29	18,15	–	12,10	13,13
A052	Y,X	18,17	16,15	11,10	11,10	8,8	14,14	32.2,30	19,15	11	14,11.3	16.2,13
A053	Y,X	17,16	18,16	10,10	11,11	11,8	15,14	31,28	15,15	10	11,10	16.2,14.2
A054	Y,X	18,15	17,14	11,10	12,10	11,8	14,14	30.2,29	23,15	10	14,11	14,13
A055	Y,X	17,15	18,14	11,9	12,12	8,8	15,11	31,30	19,14	10	14,10	15.2,14
A056	X,X	17,17	19,16	11,9	12,12	8,8	16,14	32.2,29	25,23	–	10,10	14.2,13.2
A057	X,X	17,16	19,17	11,9	11,10	9,8	14,10	29,29	19,14	–	11.3,10	13,13
A058	Y,X	17,15	18,17	12,9	12,12	10,9	10,10	31,31	19,13	10	11.3,10	17.2,14
A059	X,X	17,15	16,14	11,10	12,10	11,9	15,14	29,29	15,13	–	12,11	15,13
A060	Y,X	16,16	18,17	13,11	10,10	11,8	16,12	31,29	15,13	10	12,11.3	15,13
A061	Y,X	18,17	17,17	11,10	12,11	11,8	15,13	30,29	15,14	10	11,10	13,13
A062	Y,X	18,17	14,14	10,9	12,11	8,8	16,10	31,29	15,14	10	11,10	16.2,14
A063	X,X	17,15	17,15	11,10	11,10	11,8	16,13	32,29	15,14	–	14,10	15.2,15.2
A064	X,X	18,17	19,14	11,9	13,10	11,8	14,13	32,30	19,16	–	14,11.3	14.2,13
A065	X,X	18,18	18,18	13,9	12,12	11,8	16,10	32.2,30	14,13	–	11.3,11	14.2,13
A066	X,X	17,15	15,14	10,9	12,12	8,8	13,12	32,32	14,14	–	14,10	15.2,13.2
A067	X,X	17,15	17,16	10,9	12,12	8,8	14,11	30,29	16,15	–	14,11.3	14,13
A068	X,X	17,16	18,17	12,9	12,12	8,8	16,16	32,29	14,14	–	14,11.3	15.2,14
A069	X,X	15,15	18,17	11,9	12,12	11,8	16,15	31,29	19,15	–	14,11	15.2,13.2
A070	Y,X	17,16	18,17	11,9	11,10	9,9	16,11	30,30	16,15	10	12,10	15.2,13
A071	Y,X	16,15	18,17	10,10	12,10	11,8	15,14	33.2,29	17,16	10	14,14	14,13.2
A072	Y,X	15,15	17,14	14,10	10,10	11,10	16,11	30,29	16,15	11	14,10	14.2,13.2
A073	Y,X	17,15	17,15	10,10	12,10	11,11	14,11	32.2,31	16,15	11	11.3,10	13.2,13
A074	X,X	17,17	17,14	14,9	12,12	9,8	16,10	32.2,29	19,14	–	11.3,9.1	15.2,14.2
A075	X,X	17,16	18,14	10,9	11,10	11,9	18,15	31,29	20,15	–	10,9.1	14,13
A076	Y,X	17,16	18,17	11,9	12,11	11,9	13,11	31,29	19,18	10	12,11	13,13
A077	Y,X	18,15	18,15	10,9	11,10	11,11	13,11	31.2,29	23,17	10	14,10	15.2,12
A078	Y,X	17,17	18,15	11,11	11,11	9,9	16,16	30,30	17,15	10	11.3,11	15.2,13
A079	Y,X	17,15	18,17	9,9	10,10	11,9	16,13	32.2,30	15,14	10	11.3,11	13.2,13.2
A080	Y,X	17,17	18,18	11,9	11,11	11,11	15,15	31,30	20,17	10	11,10	14.2,13
A081	Y,X	17,17	17,14	13,12	12,10	11,11	16,16	32.2,30	17,15	10	14,10	16.2,13
A082	Y,X	16,15	17,16	10,9	13,12	9,8	16,13	33.2,30	16,15	10	12,12	15.2,13.2
A083	Y,X	16,15	18,17	11,9	11,11	11,8	15,14	31.2,31	16,15	11	15,10	15.2,14.2
A084	Y,X	17,16	18,18	12,11	12,10	9,8	14,11	31.2,29	17,15	10	14,12	13.2,13
A085	X,X	17,17	18,17	12,9	10,10	9,9	16,12	31.2,30	15,13	–	10,10	15.2,15.2
A086	Y,X	18,16	17,16	12,9	11,11	8,8	16,14	31,30	16,16	11	14,11.3	15.2,13
A087	Y,X	16,15	17,15	10,9	13,12	11,10	14,11	31,30	16,16	10	11.3,11	13.2,13
A088	X,X	17,16	18,17	13,9	11,10	9,8	15,11	30,30	12,12	–	14,10	13.2,13
A089	X,X	16,15	19,14	13,10	12,11	8,8	16,14	31,28	23,15	–	11,10	14.2,13
A090	Y,X	15,15	18,14	10,9	11,11	8,8	16,14	32.2,28	15,15	10	10,10	14.2,13
A091	Y,X	18,16	18,17	9,9	12,12	11,8	16,14	32.2,29	19,13	10	12,11.3	15,14
A092	X,X	17,15	19,15	11,9	12,11	11,8	15,12	32.2,31	20,15	–	11.3,10	16,14
A093	X,X	18,16	18,17	11,9	12,11	9,8	14,10	31,29	19,19	–	11.3,10	15.2,13
A094	Y,X	18,17	18,15	9,9	13,10	8,8	14,10	30,29	19,15	11	14,11.3	14,13
A095	Y,X	16,16	18,16	9,9	12,11	11,8	15,14	32.2,29	23,16	10	14,11	16.2,13
A096	X,X	17,15	18,17	10,9	12,12	8,8	14,11	29,29	19,13	–	12,10	17.2,14
A097	Y,X	17,15	19,19	11,11	12,10	10,9	14,11	32.2,29	15,14	9	14,10	13,13
A098	Y,X	17,15	17,14	9,9	12,12	10,8	15,11	31,30	15,15	10	12,11	17.2,16.2
A099	Y,X	16,15	18,17	12,10	12,11	11,8	16,10	31,28	22,18	11	12,11	14,13
A100	X,X	17,16	19,17	9,9	12,12	11,11	15,14	32.2,30	25,23	–	12,10	14,13.2
A101	X,X	17,16	17,14	11,11	12,10	9,9	18,11	32,30	18,14	–	11.3,11	15.2,13
A102	X,X	15,15	18,17	10,9	11,10	11,11	13,10	31.2,29	23,17	–	14,12	16.2,15.2
A103	Y,X	17,16	18,16	10,9	11,10	8,8	13,10	33.2,30	19,14	10	14,13	15.2,13
A104	Y,X	18,16	17,15	9,9	10,10	9,9	14,12	32.2,28	19,14	10	11.3,11	15.2,13
A105	Y,X	17,15	18,18	10,9	13,10	11,8	14,11	34.2,33.2	17,17	10	12,12	13,13
A106	Y,X	17,17	16,14	11,10	12,12	9,8	16,13	31,30	19,17	10	14,12	15.2,15.2
A107	Y,X	17,16	17,15	12,9	12,11	11,9	13,11	31,30.2	17,14	10	11.3,11	14.2,13
A108	Y,X	17,15	18,15	11,10	12,10	11,11	16,14	34.2,32.2	20,18	9	12,10	14.2,13
A109	Y,X	17,15	19,18	13,10	13,12	9,8	16,15	32.2,32	17,15	10	14,11	14.2,13.2
A110	Y,X	17,16	18,17	13,11	12,11	11,8	16,16	33.2,32.2	19,15	10	11.3,10	14,13
A111	Y,X	18,17	18,16	12,11	12,12	11,9	14,14	31,29	17,15	10	13,12	13,13
A112	Y,X	18,16	18,17	11,9	13,11	11,8	16,13	30,29	17,16	10	14,12	14.2,13
A113	Y,X	17,15	17,17	11,9	10,10	9,9	16,10	30,30	16,15	10	14,11	15,13
A114	Y,X	18,16	18,17	13,9	12,11	9,8	18,16	30,29	18,11	10	11.3,10	15,13
A115	Y,X	16,15	17,14	12,11	12,11	9,8	15,10	30,28	18,15	11	14,11	14.2,13.2
A116	X,X	17,16	14,14	11,10	11,10	11,9	14,13	33.2,31	17,16	–	12,11.3	15.2,14.2
A117	Y,X	17,16	17,17	13,10	11,10	11,9	15,14	30,30	16,13	10	14,10	14.2,14
A118	Y,X	16,16	18,17	11,10	11,10	8,8	16,15	32.2,29	15,13	10	11.3,10	14.2,14
A119	Y,X	17,17	18,14	13,9	12,12	9,9	16,11	31,30	18,15	10	11,10	14.2,14.2
A120	Y,X	17,17	17,14	12,12	11,10	11,9	18,16	33.2,30	23,18	9	11,10	16.2,14
A121	X,X	17,15	15,14	13,9	10,9	9,8	16,15	31,30	18,17	–	11,10	15.2,13
A122	X,X	17,15	18,17	11,9	13,10	11,11	16,11	31.2,29	17,16	–	11,10	15.2,13
A123	Y,X	17,14	18,14	10,9	12,9	11,9	14,11	30,29	15,14	11	11,10	14.2,14
A124	Y,X	17,16	18,14	10,9	11,10	11,11	15,13	31,30.2	18,15	10	11.3,10	15,13
A125	Y,X	17,15	18,16	10,10	10,10	9,8	15,10	30,30	17,15	11	14,11.3	15.2,13
A126	Y,X	17,17	18,14	12,9	10,10	9,9	18,14	29,29	18,14	11	14,11	15.2,14
A127	Y,X	17,15	18,17	10,10	12,12	9,8	18,11	31,28	19,18	10	13,11	15.2,14
A128	X,X	17,16	18,17	13,12	12,11	11,8	18,13	29,29	18,17	–	12,11	15.2,13
A129	X,X	18,16	18,17	13,10	12,12	8,8	15,10	32.2,29	23,16	–	13,12	15.2,13
A130	X,X	17,16	17,16	11,10	11,11	11,8	15,14	30,29	18,17	–	14,11.3	15.2,12
A131	Y,X	17,16	18,18	12,11	12,10	8,8	13,12	29,29	18,15	9	14,10	13,13
A132	Y,X	16,16	17,14	11,11	11,11	9,8	17,14	30.2,30	18,15	11	14,12	15.2,12
A133	X,X	17,15	18,18	11,10	12,10	8,8	15,14	32.2,29	18,16	–	14,12	15.2,14.2
A134	X,X	17,17	18,17	11,9	12,11	11,8	13,11	33.2,29	16,15	–	11.3,9.1	15.2,14.2
A135	Y,X	18,17	17,17	10,9	12,10	11,9	16,14	35.2,29	15,15	9	12,11.3	15.2,13
A136	Y,X	18,17	18,17	10,9	10,10	11,9	15,13	35.2,32.2	15,15	9	10,9.1	15.2,13
A137	Y,X	17,16	18,14	13,11	10,9	9,8	15,13	30.2,30	16,12	11	14,11.3	14.2,14
A138	Y,X	18,17	17,16	11,11	10,10	11,9	16,15	33.2,30	18,15	10	14,10	14,14
A139	X,X	16,16	17,16	12,10	12,11	11,11	13,11	32,31	16,12	–	11.3,11.3	15,13
A140	X,X	17,17	17,14	9,9	11,10	11,9	15,13	35.2,32.2	15,15	–	11,10	13,13
A141	Y,X	17,16	14,14	14,10	13,10	8,8	17,13	31.2,31	17,14	9	11,10	15.2,13
A142	Y,X	17,15	18,17	11,10	11,10	9,8	14,11	30,29	15,15	11	12,9.1	16.2,15.2
A143	Y,X	17,15	17,14	12,9	11,10	11,8	16,10	30,29	19,15	11	14,11	16.2,15.2
A144	Y,X	17,15	17,17	12,9	10,10	11,9	16,15	30,29	16,15	10	14,11	15,13.2
A145	Y,X	17,17	17,16	9,9	9,9	9,8	16,13	32.2,32	20,15	10	11.3,11	14,13
A146	Y,X	18,17	17,14	12,9	11,11	9,8	16,12	32.2,29	16,14	10	12,10	14,13
A147	Y,X	17,16	18,18	9,9	12,12	9,8	15,11	31.2,31	15,14	10	11,11	15.2,14
A148	Y,X	17,16	16,14	10,10	11,9	11,8	15,11	29,29	18,11	10	12,10	15.2,13
A149	X,X	16,15	17,17	11,11	12,11	9,8	16,13	31.2,29	17,14	–	14,10	16.2,14
A150	X,X	17,15	18,15	11,9	10,10	11,8	13,10	31,29	18,14	–	14,11	16.2,14.2
A151	Y,X	16,15	18,18	10,9	13,10	8,8	12,11	29,29	16,15	10	14,11	15.2,14
A152	Y,X	17,15	17,14	9,9	11,11	11,8	15,13	30,30	17,15	11	14,14	14.2,13
A153	Y,X	18,16	17,16	11,9	12,10	8,8	13,10	32.2,30	19,13	10	14,10	15.2,14
A154	X,X	17,17	16,14	13,9	9,9	9,8	16,16	31,30	16,15	–	12,11	15.2,13.2
A155	Y,X	18,16	19,18	11,11	12,11	11,8	16,15	31.2,30.2	17,15	10	11.3,10	16.2,13
A156	X,X	18,17	14,14	13,12	11,10	9,8	15,14	32,31.2	16,15	–	10,10	15,13.2
A157	Y,X	18,18	17,14	12,10	10,10	11,9	13,13	32.2,31	20,13	10	11.3,10	15.2,14.2
A158	Y,X	17,16	18,14	11,11	12,11	9,8	12,10	31.2,31.2	15,13	11	11,10	15.2,14
A159	Y,X	17,16	16,14	11,11	10,10	11,9	15,10	29,29	19,17	9	14,11.3	15.2,14
A160	Y,X	16,15	18,18	13,10	12,10	11,8	10,10	33.2,31	14,14	9	12,10	13,13
A161	Y,X	18,16	17,14	10,9	11,10	11,11	14,13	31,31	17,15	9	11,11	14.2,13
A162	Y,X	17,15	18,18	13,12	12,11	11,9	14,11	34.2,29	15,14	10	14,11	15.2,14
A163	Y,X	18,17	18,14	9,9	12,10	8,8	11,10	30,29	16,14	10	12,11	13,13
A164	X,X	17,15	19,18	11,10	12,10	11,8	16,16	32.2,30.2	17,15	–	13,11	16.2,15
A165	X,X	15,15	18,16	13,11	12,10	11,9	14,11	31.2,29	16,15	–	13,12	14.2,13
A166	Y,X	17,16	17,14	10,10	12,10	9,8	18,10	33.2,29	15,15	10	12,10	16.2,13
A167	Y,X	18,17	17,17	13,10	12,10	11,11	16,10	29,29	14,14	10	14,14	15.2,15.2
A168	X,X	16,15	18,17	13,13	12,12	9,8	16,13	33.2,32.2	19,14	–	11,10	13,13
A169	Y,X	17,16	18,17	9,9	12,10	9,8	18,18	31.2,31.2	16,16	10	14,11	14.2,13
A170	Y,X	17,15	18,17	10,9	13,10	8,8	18,16	31.2,31	16,14	10	14,12	15,14.2
A171	Y,X	18,17	17,17	10,10	12,10	12,11	15,13	32.2,32.2	23,19	10	14,14	15.2,13
A172	Y,X	17,15	16,16	10,9	11,10	8,8	13,11	31.2,30	18,15	10	11.3,11	15.2,13
A173	X,X	16,16	17,14	11,9	11,11	11,11	15,15	30.2,29	18,18	–	11.3,10	13,12
A174	Y,X	18,15	18,17	13,9	11,11	11,8	15,14	33.2,30	17,15	10	10,10	13,13
A175	Y,X	17,16	18,14	13,13	10,10	9,8	16,12	32.2,31	15,15	9	11.3,11	15.2,14.2
A176	X,X	17,17	17,17	13,10	12,8	9,8	13,11	31,31	16,15	–	14,14	13,12
A177	Y,X	16,15	17,14	12,11	11,10	11,11	14,14	32.2,29	18,14	10	11,11	14,13
A178	X,X	18,15	17,14	10,9	12,12	8,8	16,10	31.2,29	15,15	–	14,10	13,13
A179	X,X	18,15	19,17	11,11	11,10	9,8	14,13	31,30	20,12	–	12,11	15,13
A180	X,X	17,16	18,16	12,9	12,9	11,8	16,15	32,29	19,15	–	11.3,11.3	15.2,14
A181	Y,X	17,17	18,18	13,11	12,10	9,8	15,13	31,31	14,13	10	11.3,11	14.2,13
A182	X,X	16,16	19,18	12,12	11,10	11,8	17,13	32.2,32.2	23,15	–	11.3,11.3	16.2,15.2
A183	X,X	17,15	17,14	10,9	10,10	8,8	16,15	31,30	17,15	–	14,12	14.2,13
A184	Y,X	18,15	18,17	12,11	12,10	11,8	14,13	32,31	20,18	10	12,12	13,12
A185	Y,X	16,16	18,16	11,10	12,9	11,11	14,12	32.2,29	17,14	11	13,11.3	13,13
A186	Y,X	17,16	18,14	11,10	10,10	9,8	13,11	32,29	15,12	10	14,11	16.2,14.2
A187	X,X	17,17	17,14	13,11	12,12	8,8	16,16	31.2,30	19,15	–	11.3,11	13,13
A188	Y,X	17,16	18,17	9,9	12,12	11,11	14,13	32,29	18,15	10	10,9.1	14.2,12
A189	X,X	16,15	17,14	11,9	10,9	11,8	12,11	31,31	16,14	–	14,12	16.2,15.2
A190	Y,X	16,15	18,18	11,10	12,9	11,11	14,14	30,29	17,16	11	11,10	14,13
A191	Y,X	15,15	19,17	11,10	11,10	8,8	18,16	32.2,32	17,15	11	14,10	15.2,15
A192	Y,X	16,16	19,17	13,9	10,10	11,8	13,12	33.2,30	15,13	11	14,10	14.2,13
A193	Y,X	16,15	18,16	13,9	12,10	9,9	15,11	31,30	22,17	10	15,12	14,12
A194	X,X	18,16	18,17	11,11	12,12	12,8	16,12	30,29	16,16	–	11.3,11	14.2,13.2
A195	Y,X	17,15	17,16	11,9	12,12	8,8	13,12	33.2,32.2	16,15	9	11.3,11	16.2,13
A196	Y,X	17,15	15,14	12,10	13,12	11,9	15,13	32.2,29	14,10	10	14,12	14,13
A197	X,X	15,15	19,18	13,13	11,10	9,9	14,13	32.2,31	16,14	–	14,11	14,14
A198	X,X	15,15	19,14	11,9	11,10	11,8	13,12	32.2,32.2	18,17	–	11.3,10	15,14
A199	X,X	16,15	16,15	11,10	12,10	8,8	15,11	31,29	19,12	–	14,14	14,14
A200	Y,X	17,16	19,16	12,11	12,11	8,8	14,10	30,29	15,15	11	12,11	14.2,13

Ϯ Sex determining STR loci.

**Table 1b:** STR allelic profiles for THO1, FGA, D22S1045, D5S818, D13S317, D7S820, SE33, D10S1248, D1S1656, DYS391 and D2S1338 in the population of Kedayan (*n* = 200).

Sample ID	Locus										
	THO1	FGA	D22S1045	D5S818	D13S317	D7S820	SE33	D10S1248	D1S1656	D12S391	D2S1338
A001	9,6	24,23	17,14	13,10	12,12	9,8	27.2,19	14,12	15,13	22,17	23,23
A002	8,7	25,23	17,14	11,11	12,8	13,11	28.2,12.2	15,13	15,13	22,19	25,18
A003	9,7	21,21	15,15	13,10	10,7	11,11	25.2,23	15,13	15,14	24,20	23,22
A004	9,9	25,22	17,17	10,10	12,8	11,10	27.2,27.2	13,13	17.3,14	20,18	20,17
A005	8,6	26,21	17,16	12,11	11,8	11,11	27.2,17	13,13	17,16	19,19	24,18
A006	9,9	22,19	17,17	10,10	11,8	12,8	28.2,27.2	14,12	17.3,14	22,17	23,20
A007	9,9	26,21	15,11	11,10	12,8	13,11	23.2,16	13,12	17.3,17	20,19	23,20
A008	9,7	27,26	16,15	11,11	8,8	12,10	19,17	13,13	16,14	20,19	24,23
A009	9,9	27.2,25	15,11	12,10	8,8	11,11	27.2,23.2	15,13	17,16	25,19	24,20
A010	9,7	24,19	15,11	11,11	13,12	11,10	28.2,19	14,13	18.3,16	20,18	23,19
A011	7,7	25,21	16,15	12,11	11,8	11,9	25.2,18	14,14	16.3,11	20,18	20,20
A012	9,9	24,23	17,15	10,10	13,11	11,10	19,17	14,13	17.3,11	18,18	24,18
A013	7,7	22,19	17,17	11,11	8,8	12,11	28.2,27.2	13,13	15,12	20,20	25,24
A014	8,7	22,22	17,15	11,11	11,8	12,11	30.2,28.2	14,13	17.3,12	20,18	24,24
A015	8,6	23,23	14,11	11,11	10,10	12,11	23,19	14,14	17.3,16	20,20	24,23
A016	9.3,9	24,21	15,11	13,11	11,8	11,11	27.2,16	14,12	16,13	22,19	24,20
A017	10,6	21,19	15,14	11,10	11,8	11,10	27.2,17	14,13	17.3,13	20,17	23,18
A018	8,8	21,19	15,15	11,10	11,8	12,11	27.2,19	14,12	17.3,11	20,17	25,19
A019	9,8	23,23	15,11	12,10	12,9	11,10	32.2,27.2	14,13	15,13	20,19	24,22
A020	9,9	22,21	16,11	11,11	13,12	11,10	22.2,19	14,12	17,13	21,20	24,23
A021	9,8	22,21	17,11	12,10	11,10	10,10	25.2,21	13,13	17.3,16	22,20	20,20
A022	8,7	22,21	11,11	12,11	12,8	11,7	31.2,21	15,13	17.3,16	22,20	24,20
A023	10,7	24,23	15,11	11,11	11,11	11,8	28.2,21	14,14	17.3,13	23,21	24,19
A024	9,7	23,22	15,11	11,10	12,11	11,8	26.2,18	15,14	17,13	21,17	23,23
A025	9,7	22,21	16,15	10,10	8,8	11,9	25.2,19	14,12	14,11	19,17	23,19
A026	9,9	23,22	15,15	12,10	12,10	12,8	29.2,19	15,13	16,15	25,17	25,23
A027	7,7	25,23	16,15	11,10	12,11	11,8	24.2,20	15,14	17,11	20,17	24,19
A028	9,6	23,22	17,16	11,10	12,9	11,11	20,17	15,14	15,11	19,17	23,19
A029	9,9	24,22	17,17	11,10	12,12	11,10	31.2,28.2	16,14	17,15	20,20	24,16
A030	9,9	22,22	16,11	10,7	11,8	12,11	27.2,16	15,14	17.3,12	20,20	24,24
A031	9,9	23,21	17,15	12,10	8,8	11,10	31.2,27.2	13,13	16,12	18,18	24,19
A032	9,9	25,23	15,15	11,10	12,9	12,9	28.2,19	15,14	14,11	17,17	19,19
A033	9,7	26,23	17,15	12,10	11,8	11,9	27.2,17	14,13	16,16	19,18	23,19
A034	9,6	24,19	15,11	13,10	10,8	11,10	25.2,21	16,13	16,12	22,18	24,22
A035	9,7	22,21	17,15	10,10	8,8	11,11	27.2,25.2	15,12	15,14	22,20	20,19
A036	9,9	23,23	16,15	14,9	12,12	11,10	24.2,19	15,14	16,12	22,18	19,18
A037	9,7	23,19	17,11	10,10	9,8	10,8	28.2,28.2	14,13	13,12	21,20	24,22
A038	9,9	19,19	17,11	13,10	9,8	11,8	28.2,19	16,14	16,12	21,18	24,21
A039	9,7	23,23	15,11	14,10	12,8	11,8	24.2,17	14,14	16,15	22,20	19,16
A040	9,8	24,21	16,15	13,9	10,8	11,10	19,17	14,14	15,14	26,19	23,16
A041	7,7	23,23	11,11	10,10	8,8	12,10	28.2,25.2	16,13	15,13	20,20	24,22
A042	9,7	22,19	17,17	12,11	12,11	11,11	31.2,26.2	15,13	17.3,15	21,19	24,23
A043	9,7	25,21	17,16	10,10	10,8	11,10	30.2,21	14,13	15,13	26,20	24,23
A044	9,6	24,21	17,17	12,11	12,12	11,10	29.2,26.2	16,14	15,11	20,20	24,24
A045	9.3,9	26,23	17,17	10,10	11,10	11,8	31.2,18	16,15	17.3,15	18,18	24,24
A046	8,7	21,19	16,11	11,10	11,8	11,10	31.2,17	16,15	13,11	25,20	26,23
A047	9,7	22,19	17,15	14,10	12,11	11,10	19,16	15,13	15,11	19,17	25,16
A048	7,6	24,22	16,15	10,10	12,11	13,11	28.2,21	16,14	15,15	25,20	24,19
A049	9.3,9	24,24	15,15	11,11	12,12	12,12	29.2,17	14,13	17.3,15	20,19	20,16
A050	9,9	23,21	15,11	11,9	8,8	12,10	27.2,17	13,13	16,12	20,18	23,22
A051	9,7	22,21	17,11	13,12	11,8	11,8	19,17	14,14	15,14	18,17	23,19
A052	10,7	25,19	17,11	11,10	12,10	11,10	27.2,27.2	16,13	16,16	22,20	23,19
A053	9,7	25,25	17,11	10,10	12,11	12,10	27.2,26.2	14,14	16,16	22,20	20,19
A054	9,6	25,21	15,11	12,11	12,8	11,10	28.2,26.2	17,15	17,13	20,18	22,19
A055	9,9	23,23	15,11	14,10	12,12	11,10	28.2,19	13,12	16,14	20,18	23,22
A056	9.3,7	23,21.2	15,11	11,10	12,8	10,10	26.2,26.2	15,14	16,11	25,20	23,22
A057	7,7	27,25	15,15	11,10	11,10	11,9	20,20	13,13	17.3,17	19,18	17,17
A058	9,7	22,22	15,15	10,9	9,8	11,8	26.2,18	17,14	17.3,16	18,18	23,18
A059	10,7	25,23	16,11	12,10	8,8	10,10	31.2,27.2	16,13	16,16	21,18	23,19
A060	9,7	23,23	15,15	10,10	12,11	11,8	27.2,26.2	16,15	17.3,16	25,23	23,23
A061	10,9	24,18.2	15,11	11,10	12,11	10,8	27.2,20	16,13	17.3,16	24,20	24,19
A062	9,9	25,23	17,16	11,10	11,10	11,10	28.2,19	14,13	16,16	20,20	19,19
A063	9,7	23,22	15,15	12,10	11,8	11,10	29.2,24.2	14,13	17,16	20,20	24,19
A064	9,7	22,12	15,11	12,11	12,11	11,11	28.2,28.2	17,14	16.3,15	20,20	20,17
A065	9,8	24,21	18,15	12,11	12,10	10,8	29.2,17	15,14	16,15	18,17	18,16
A066	9,8	24,23	18,17	12,10	11,10	11,10	29.2,27.2	13,13	18,17	20,20	23,19
A067	9,6	25,24	14,11	12,10	8,8	12,9	28.2,19	14,13	14,12	25,20	23,23
A068	7,7	23,23	15,11	11,10	10,8	11,10	28.2,19	15,14	18,17.3	22,17	24,19
A069	9,9	24,24	15,15	10,10	12,8	11,8	27.2,17	14,13	16,13	22,17	24,17
A070	9,8	22,19	16,15	12,10	12,10	11,11	28.2,17	14,13	18,16	20,17	24,18
A071	9.3,7	25,21	16,15	11,10	12,8	12,11	26.2,25.2	15,13	16,14	23,20	23,19
A072	9.3,9	23,23	17,15	11,11	10,10	12,10	29.2,26.2	13,12	16,14	22,22	24,23
A073	9,9	23,21	17,15	13,11	10,10	11,11	29.2,19	13,12	14,14	23,21	24,17
A074	7,7	25,23	15,15	11,11	12,10	11,11	20,19	15,14	17.3,16	22,22	19,19
A075	9,9	22,19	15,11	11,11	12,9	9,8	27.2,16	14,13	16,11	18,17	23,19
A076	9,9	23,22	17,11	12,11	12,8	11,10	27.2,19	15,14	15,11	19,17	26,19
A077	9,8	25,22	15,11	13,12	8,8	13,11	29.2,27.2	17,14	17.3,14	26,20	24,22
A078	9,8	21,21	15,15	10,10	11,8	11,10	25.2,21	14,14	13,12	18,17	24,23
A079	9,7	25,21	15,11	11,11	12,10	11,11	29.2,17	14,13	15,11	20,17	17,16
A080	9,9	23,21	15,15	13,10	10,8	11,11	27.2,24.2	16,13	15,14	19,19	24,24
A081	9,6	22,21	17,11	12,11	11,10	11,10	23,19	17,16	12,11	19,18	26,24
A082	8,8	24,21	15,15	11,11	11,11	10,8	29.2,16	15,13	17.3,15	20,18	24,19
A083	9,6	26,22	16,15	12,10	12,10	11,9	29.2,28.2	15,14	17,15	24,20	17,16
A084	9,7	23,22	15,14	10,10	10,9	11,9	28.2,19	16,13	15,14	19,17	20,18
A085	7,7	21,20	15,15	12,9	13,8	11,10	27.2,22	13,13	17,13	22,20	23,20
A086	9.3,9	21,20	16,15	12,10	11,10	9,8	28.2,17	14,14	17.3,11	25,19	24,23
A087	9,7	22.2,19	17,15	13,13	12,8	12,10	27.2,19	14,13	16,14	22,17	24,23
A088	11,7	21,19	17,15	11,11	10,8	10,8	26.2,21	15,13	16,14	21,19	25,23
A089	9.3,7	24,21	16,11	13,11	12,9	11,10	27.2,19	14,13	13,12	24,21	23,19
A090	9.3,7	24,21	17,16	11,10	11,9	11,10	29.2,17	16,13	14,12	22,19	23,18
A091	9,7	25,22	17,11	12,11	12,12	12,10	24.2,19	14,13	16,14	24,18	24,17
A092	10,9	25,22	16,16	10,10	12,10	12,11	30.2,19	15,14	16,15	20,19	24,24
A093	9,9	24,24	17,17	12,10	12,11	12,8	26.2,19	13,12	13,11	19,18	20,17
A094	7,6	25,23	17,17	12,10	11,10	10,10	27.2,17	14,13	16,14	23,20	17,16
A095	9,7	25,21.2	17,15	11,10	12,12	10,10	29.2,26.2	16,14	16,16	22,20	23,19
A096	10,9	25,22	16,11	12,11	12,12	11,10	24.2,18	14,13	16,14	24,22	19,18
A097	9,6	25,24	16,15	12,9	12,12	12,10	26.2,18	14,14	16,15	21,18	18,17
A098	9,9	24,21	17,15	11,10	12,11	11,10	26.2,18	14,14	15,13	22,19	19,18
A099	9,7	25,21.2	15,11	10,10	12,11	11,11	29.2,27.2	15,15	16,11	25,22	23,19
A100	9,7	25,23	15,11	11,11	12,12	11,10	26.2,26.2	15,14	13,11	25,19	23,19
A101	8,7	23,23	16,15	11,10	12,11	11,10	28.2,27.2	14,13	16,11	22,17	24,17
A102	10,8	23,22	15,11	13,11	8,8	13,11	29.2,22.2	14,14	17.3,14	22,19	24,22
A103	9,7	22,21	11,11	14,10	11,9	11,10	27.2,21	15,14	16,15	20,19	24,23
A104	9,6	23,21	15,15	10,10	12,8	11,11	29.2,27.2	17,13	17.3,14	24,20	25,24
A105	10,7	24,22	15,14	10,10	10,9	10,9	27.2,18	14,13	17.3,11	20,19	23,23
A106	10,8	24,22	15,11	11,10	9,8	11,11	25.2,23	15,13	14,13	24,20	23,19
A107	9,9	24,22	17,15	10,9	11,9	11,9	27.2,12.2	15,14	17.3,14	20,20	25,24
A108	9,8	23,22	17,15	10,10	12,10	11,8	21,18	15,14	17,16	24,18	20,19
A109	9,7	24,21	16,11	11,11	10,9	12,8	29.2,27.2	14,13	14,11	20,19	26,23
A110	8,7	25,22	17,15	11,10	9,8	11,11	29.2,25.2	14,12	17.3,11	23,21	23,20
A111	9,9	21,21	17,11	13,11	8,7	11,11	30.2,25.2	13,13	17.3,14	20,19	23,23
A112	7,7	24,24	15,15	11,10	11,8	12,11	30.2,17	15,13	14,12	22,19	23,22
A113	9,9	22,21	11,11	12,12	11,11	12,11	20,19	14,14	17.3,11	19,19	24,23
A114	8,7	21,19	16,11	11,10	12,9	11,9	27.2,27.2	14,12	16,14	19,17	23,23
A115	9,7	26,25	15,15	12,12	12,11	12,11	26.2,25.2	16,13	17,12	20,17	23,20
A116	9,8	25,19	15,15	11,10	11,8	13,11	19,17	14,14	16,15	20,18	24,23
A117	9,9	23,21	17,15	13,13	11,11	12,11	26.2,22.2	16,12	13,11	19,17	23,18
A118	8,7	26,21	17,16	12,10	12,10	12,11	31.2,19	15,14	15,15	19,17	24,23
A119	9.3,7	23,19	16,15	10,9	12,12	11,11	29.2,20	15,12	15,13	19,19	24,17
A120	9,7	25,23.2	11,11	11,10	12,11	11,8	28.2,20	14,14	14,13	24,20	23,20
A121	9,9	26,20	17,17	11,10	11,8	11,11	30.2,18	13,13	16,11	19,18	26,24
A122	9.3,9	24,21	16,15	11,10	12,8	11,11	27.2,17	14,13	15,15	19,18	23,17
A123	9.3,9	25,22	15,11	12,10	12,8	12,10	28.2,17	14,14	16.3,16	20,17	23,16
A124	8,7	27,21	14,11	10,10	12,8	10,8	27.2,26.2	17,13	15,13	20,17	23,23
A125	9,7	27.2,26	16,11	11,10	12,12	13,10	23.2,21.2	14,13	15,13	19,19	20,18
A126	8,7	23.2,22	17,15	12,11	12,11	11,11	28.2,26.2	14,13	14,11	24,18	20,20
A127	9.3,8	25,24	15,15	12,9	10,8	12,11	27.2,19	14,14	15,14	20,17	23,17
A128	9,9	24,24	15,14	13,12	11,8	12,11	21,17	14,13	17,12	18,17	23,20
A129	9,9	25,24	16,15	11,10	12,9	12,10	24.2,16	15,14	17,16	19,17	24,23
A130	9,7	24,22	16,15	11,11	12,12	10,8	17,16	14,13	15,12	20,18	23,20
A131	9,8	26,22	17,11	13,11	12,8	11,10	24.2,24.2	14,14	18,12	20,17	24,19
A132	9,6	26,22	15,11	12,12	12,8	12,11	24.2,23.2	13,13	16,14	21,20	22,19
A133	9,9	24,21	17,14	13,10	12,8	11,9	24.2,19	14,14	17.3,17	18,17	24,23
A134	8,7	23,23	15,14	11,10	12,12	13,12	29.2,19	13,13	17.3,15	19,17	25,20
A135	9,9	24,23	15,11	10,10	11,11	11,11	25.2,23.2	14,13	16.3,14	18,18	22,17
A136	9,9	23,19	15,15	11,10	12,9	12,11	28.2,16	14,13	16,14	18,17	23,23
A137	9,7	24,21	15,11	11,11	14,9	10,10	28.2,28.2	15,14	17.3,14	20,18	23,23
A138	8,7	25,22.2	17,11	11,10	14,8	11,11	29.2,16	14,14	12,11	22,19	24,17
A139	7,6	25,23	15,11	12,10	11,8	11,10	27.2,26.2	15,13	17.3,17	19,19	24,23
A140	9,7	22,21	15,15	11,11	12,9	12,10	28.2,16	15,14	17.3,13	19,18	23,22
A141	9.3,9	21,19	15,15	12,10	9,8	11,10	19,19	14,13	16.3,16	18,18	22,17
A142	9,7	24,21	11,11	11,10	11,9	10,10	28.2,28.2	15,14	15,15	18,18	19,17
A143	7,6	25,21.2	15,11	11,10	10,8	12,11	19,17.2	17,14	17.3,11	24,19	17,16
A144	9,7	22,21	14,11	15,13.2	9,7	22,21	11,11	12,12	11,11	8,8	27.2,19
A145	9,9	23,21	15,15	10,9	11,8	13,10	28.2,24.2	15,14	12,11	18,18	22,16
A146	9,7	25,19	16,11	11,10	12,11	10,9	27.2,23	14,13	17.3,17.3	23,19	17,17
A147	9,8	22,19	17,15	12,10	12,8	11,8	26.2,21	14,14	11,11	18,18	24,19
A148	9,6	23,21	17,16	11,11	11,11	11,11	27.2,26.2	16,14	15,13	20,17	22,20
A149	10,7	26,22	11,11	10,10	12,11	11,10	29.2,19	14,12	17,13	22,19	19,19
A150	9,7	23,21	14,11	12,11	11,11	11,11	27.2,27.2	16,13	15,12	19,18	23,18
A151	9,9	22,21	11,11	13,9	12,10	11,8	25.2,21.2	14,14	16,16	22,19	23,17
A152	9,7	26.2,21	15,11	13,10	12,11	11,8	28.2,26.2	13,13	16.3,14	22,19	22,20
A153	9,7	24,21.2	15,15	12,9	8,8	11,10	26.2,23	13,13	16,11	19,17	23,20
A154	9,8	24,21	15,11	9,9	12,9	13,10	26.2,17	16,15	16,15	19,17	20,18
A155	9,8	21,21	17,11	13,11	12,12	9,8	27.2,18	13,13	16,15	23,17	23,23
A156	7,6	24,22	16,15	11,11	10,9	11,9	31.2,25.2	15,13	17.3,15	23,19	19,18
A157	9.3,9	24,24	16,15	13,12	12,9	12,8	30.2,27.2	15,14	17,16	23,20	23,18
A158	9,7	25,22	17,15	12,11	11,10	11,10	33.2,29.2	15,13	16,13	22,20	24,23
A159	9,7	23,21	17,11	11,11	11,10	10,9	19,16	14,13	18,16	21,20	19,19
A160	8,7	22,21	16,15	10,10	12,12	11,10	27.2,17	16,14	15,11	20,20	22,20
A161	8,7	21,18	14,11	12,11	12,12	13,9	31.2,29.2	15,15	13,13	20,17	26,23
A162	9,8	19,19	17,11	12,10	12,11	11,9	29.2,21	14,13	15,15	19,18	24,20
A163	9,9	24.2,24	15,15	12,11	8,8	11,9	19,19	15,13	12,11	20,19	24,23
A164	9,7	26,22	16,15	11,7	11,11	12,10	28.2,19	14,13	16,16	19,18	24,23
A165	7,6	24,21	15,11	12,10	12,8	11,8	26.2,25.2	14,13	16,13	19,18	19,17
A166	9.3,7	24,19	15,11	12,10	12,12	12,10	19,17	14,14	17.3,17.3	20,19	22,16
A167	9,7	27,25	16,15	12,10	11,10	11,8	28.2,27.2	15,13	16,15	25,24	23,20
A168	9.3,9	23,23	16,15	11,10	12,11	12,11	25.2,24.2	15,14	15,15	22,19	23,23
A169	9,9	23,21	15,15	12,12	10,10	11,11	24.2,19	14,13	16,12	19,18	24,19
A170	9,9	23,22	15,11	12,9	10,9	11,11	24.2,23.2	14,14	16,12	19,18	19,16
A171	9,8	24,22	17,17	13,12	12,11	11,10	28.2,28.2	14,13	16,15	20,18	24,24
A172	9,9	25,23	17,15	10,10	12,10	11,8	27.2,17	14,14	15,12	20,18	20,18
A173	8,7	22,21	15,11	11,10	12,12	10,8	27.2,16	17,14	13,12	20,17	23,23
A174	9,8	25,21	15,14	10,10	12,11	12,9	25.2,22.2	15,13	17.3,11	20,18	19,19
A175	9.3,7	24,22	17,15	11,11	11,10	11,8	26.2,17	16,13	18,15	20,18	24,18
A176	9,7	22,22	15,15	13,10	12,11	9,8	19,17	13,13	15,12	22,17	24,23
A177	7,6	21,21	17,16	10,10	12,11	11,10	29.2,28.2	14,13	17,16	24,19	23,20
A178	9,7	24,23	17,11	12,11	12,11	12,11	28.2,26.2	13,13	16,13	19,19	23,22
A179	9,8	25,21	17,11	11,10	11,8	11,10	30.2,22	16,13	18,17.3	19,18	23,17
A180	9,7	26,25	17,16	11,10	12,8	13,11	29.2,17	14,13	16,14	18,17	24,23
A181	10,6	22,21	17,15	12,11	9,8	13,11	27.2,26.2	14,13	17,13	20,19	24,18
A182	8,7	23,23	17,15	10,10	12,11	12,12	25.2,18	15,14	12,11	22,18	24,23
A183	9,9	23,23	15,11	12,10	12,12	11,10	17,17	14,14	15,15	20,17	23,16
A184	9,8	25,21	16,11	10,9	11,11	13,11	30.2,22	13,13	17.3,17.3	19,18	17,16
A185	9,9	27,23	15,15	11,10	12,11	12,11	25.2,20	15,15	17,11	17,17	24,23
A186	10,7	22,21	11,11	11,11	10,10	11,11	20,17	16,14	16,16	20,18	19,19
A187	7,6	22,21	16,15	12,10	12,8	11,10	27.2,17	16,14	16,13	20,17	23,16
A188	9,6	24,22	15,15	13,11	12,10	11,8	24.2,21	14,13	16,15	22,20	22,16
A189	9,7	25,22	16,15	13,11	10,9	11,11	27.2,25.2	15,13	17,15	22,17	24,19
A190	9,9	25,23	16,15	11,10	12,11	11,10	28.2,18	14,13	15,13	20,17	24,19
A191	7,7	23,21	15,11	11,10	12,12	12,10	26.2,23.2	14,13	15,14	21,17	22,16
A192	9.3,9	26.2,21	15,11	13,11	11,10	10,8	28.2,26.2	14,13	17,13	23,21	18,16
A193	10,7	26,22	15,15	13,12	9,8	8,8	28.2,19	14,13	16,16	21,19	18,17
A194	7,7	25,21	16,11	10,10	11,10	11,10	25.2,23	14,13	16,15	20,18	23,23
A195	9,7	25,20	17,11	10,10	8,8	12,10	20,14	17,16	16,11	20,18	19,17
A196	9,7	23,19	17,11	13,10	8,8	11,11	25.2,25.2	14,13	15,15	22,20	23,22
A197	7,6	24,24	11,11	10,10	12,9	11,10	26.2,23.2	14,13	17.3,16	21,19	23,23
A198	10,9.3	24,21	17,17	11,10	11,8	11,10	26.2,22	15,14	15,15	21,19	23,16
A199	8,7	23,19	15,11	12,11	12,8	10,10	28.2,22	15,13	16,15	22,19	23,23
A200	9,9	27,21	15,15	12,11	11,11	12,8	29.2,15	14,12	16,15	18,18	24,19

**Table 2:** HWE analysis for the 21 autosomal STR loci in the Kedayan population (*n* = 200).

*Parameters	CSF1PO	D10S1248	D12S391	D13S317	D16S539	D18S51	D19S433	D1S1656	D21S11	D22S1045	D2S1338	D2S441	D3S1358	D5S818	D7S820	D8S1179	FGA	SE33	TH01	TPOX	vWA
Hobs	0.720	0.715	0.825	0.830	0.680	0.870	0.830	0.910	0.750	0.725	0.805	0.875	0.825	0.850	0.850	0.835	0.745	0.620	0.735	0.685	0.790
He	0.698	0.700	0.779	0.848	0.656	0.865	0.839	0.921	0.721	0.731	0.814	0.854	0.842	0.817	0.860	0.856	0.771	0.707	0.778	0.723	0.727
*p*-value	0.597	0.910	0.875	0.760	0.391	0.719	0.069	0.263	0.849	0.400	0.837	0.721	0.720	0.382	0.023	0.072	0.762	0.020	0.314	0.030	0.259

⁎ Hobs; observed heterozygosity, He; expected heterozygosity, *p*-value; probability values of exact tests for HWE.

**Table 3:** LD analysis for pairs of 21 autosomal STR loci. Several pairs of loci located on the same chromosome are in LD at significance level *p* < 0.05. There was no significant LD after applying Bonferroni correction (*p* <0.002).

LOCUS	THO1	D13S1358	VWA	D21S11	TPOX	D1S1656	D12S391	SE33	D10S1248	D22S1045	D19S433	D8S1179	D2S1338	D2S441	D18S51	FGA	D16S539	CSF1PO	D13S317	D5S818	D7S820
THO1	*	0.0450	–	–	–	–	–	–	–	–	–	–	–	–	–	–	–	–	–	–	–
D13S1358		*	–	–	0.0284	–	0.0127	–	–	–	–	–	–	–	–	0.0147	–	0.0372	–	–	–
VWA			*	–	–	–	0.0108	0.0108	–	–	–	–	–	0.0235	–	–	–	–	–	–	0.0010
D21S11				*	–	–	–	–	–	0.0156	–	0.0313	0.0166	–	–	–	–	–	–	–	–
TPOX					*	–	–	–	–	–	–	0.0420	–	–	–	–	–	–	–	0.0411	–
D1S1656						*	0.0020	–	0.0284	–	–	–	0.0254	–	–	–	–	–	0.0459	–	–
D12S391							*	–	–	0.0420	–	–	–	–	–	–	–	–	0.0323	0.0049	0.0127
SE33								*	0.0078	–	–	–	0.0010	–	–	–	–	–	–	0.0127	0.0225
D10S1248									*	–	0.0010	–	0.0176	–	0.0176	–	–	–	–	–	–
D22S1045										*	0.0020	–	0.0029	–	–	–	–	–	–	–	0.0010
D19S433											*	–	0.0039	–	0.0010	–	–	–	–	–	–
D8S1179												*	–	–	–	–	–	–	–	–	–
D2S1338													*	–	–	–	–	–	–	0.0068	–
D2S441														*	–	–	–	–	–	–	–
D18S51															*	–	–	–	–	–	–
FGA																*	–	–	–	–	–
D16S539																	*	–	–	–	–
CSF1PO																		*	–	0.0391	–
D13S317																			*	–	–
D5S818																				*	0.0264
D7S820																					*

## 2 Experimental design, materials, and methods

### 2.1 Ethics statement

Prior to this research, an ethics permit (cert no: USM/JEPeM/15,100,366) was obtained from the Human Ethical Committee, Universiti Sains Malaysia.

### 2.2 Sample collection

Two hundred blood samples were collected from unrelated healthy representatives (128 males and 72 females) of the Kedayan. Sampling locations were Sipitang and Labuan Island of Sabah and Lawas in Sarawak. Participants were interviewed and their written informed consent was obtained before blood sampling. Sampling criteria include three generations of un-admixed history with other ethnicities and that all participants should have no history of illness and are unrelated to each other. Other detailed descriptions of materials, methods, experimental work and data analyses are given elsewhere in Ref [1].

### 2.3 Cell Lysis and STR genotyping

Collected blood samples on FTA cards (Whatman, UK) were punched and treated with 3 μl of Prep-n-Go™ Lysis Buffer (Thermo Fisher Scientific, Inc., Waltham, MA, USA). The lysates were then mixed with reaction mixture contain in Globalfiler™ Express PCR Amplification kit (Thermo Fisher Scientific, Inc., Waltham, MA, USA) and amplification was carried out on a GeneAmp^Ⓡ^ PCR System 9700 Thermal Cycler (Life Technologies, Foster City, CA). The thermal cycling parameters were as followed; initial denaturation at 95 °C for 1 min; 27 cycles at 94 °C for 3 s and 60 °C for 30 s, followed by final extension at 60 °C for 8 min. As a quality assurance, Control DNA 007 included in the commercial kit (Thermo Fisher Scientific, Inc., Waltham, MA, USA) was also added in each PCR reaction set-up. Post PCR procedure involved separation of STR specific amplicons, allelic ladder and internal Size Standard dye of 600 LIZ™ v2 (Thermo Fisher Scientific, Inc., Waltham, MA, USA) using capillary electrophoresis in an ABI 3500xl Genetic Analyzer (Thermo Fisher Scientific, Inc., Waltham, MA, USA), according to manufacturer's guidelines. GeneMapper^Ⓡ^ ID-X software version 1.4 (Thermo Fisher Scientific, Inc., Waltham, MA, USA) was used for determining STR allele calls.

### 2.4 Data analysis

HWE and LD estimations were carried out using Arlequin software version 3.5 and both analyses were considered significant at the *p-value* <0.05 [14]. The *p*-value for HWE tests was then adjusted to <0.002 using Bonferroni correction [15]. This *p-value* <0.002 was obtained by dividing the standard *p-value* (0.05) with total number of tested loci (i.e. 21 locus). Similarly, standard significant level for deviation from LD between pair of STR loci (*<* 0.05) was also adjusted to *p*<0.0002 (0.05/231, where 231 is the total combinations of STR loci) using Bonferroni correction.

## Declaration of Competing Interest

The authors declare that they have no known competing financial interests or personal relationships which have, or could be perceived to have, influenced the work reported in this article.
